# *Fusarium graminearum* in Stored Wheat: Use of CO_2_ Production to Quantify Dry Matter Losses and Relate This to Relative Risks of Zearalenone Contamination under Interacting Environmental Conditions

**DOI:** 10.3390/toxins10020086

**Published:** 2018-02-17

**Authors:** Esther Garcia-Cela, Elsa Kiaitsi, Michael Sulyok, Angel Medina, Naresh Magan

**Affiliations:** 1 Applied Mycology Group, Environment and AgriFood Theme, Cranfield University, Cranfield Beds. MK43 0AL, UK; m.e.garcia-cela@cranfield.ac.uk (E.G.-C.); Elsa.Kiaitsi@cranfield.ac.uk (E.K.); a.medinavaya@cranfield.ac.uk (A.M.); 2 Centre for Analytical Chemistry, Department of Agrobiotechnology (IFA-Tulln), University of Natural Resources and Life Sciences Vienna (BOKU), Konrad Lorenz str. 20, A-3430 Tulln, Austria; michael.sulyok@boku.ac.at

**Keywords:** *Fusarium graminearum*, mycotoxins, water activity, temperature, respiration rates, risk, cereals, The relationship between quality loss in stored wheat under different environmental conditions due to *F. graminearum* colonization has shown that very small changes in dry matter loss may lead to ZEN levels above the EU legislative limits. This data could be used to help develop a post-harvest Decision Support System for better management during grain storage.

## Abstract

Zearalenone (ZEN) contamination from *Fusarium graminearum* colonization is particularly important in food and feed wheat, especially during post-harvest storage with legislative limits for both food and feed grain. Indicators of the relative risk from exceeding these limits would be useful. We examined the effect of different water activities (a_w_; 0.95–0.90) and temperature (10–25 °C) in naturally contaminated and irradiated wheat grain, both inoculated with *F. graminearum* and stored for 15 days on (a) respiration rate; (b) dry matter losses (DML); (c) ZEN production and (d) relationship between DML and ZEN contamination relative to the EU legislative limits. Gas Chromatography was used to measure the temporal respiration rates and the total accumulated CO_2_ production. There was an increase in temporal CO_2_ production rates in wetter and warmer conditions in all treatments, with the highest respiration in the 25 °C × 0.95 a_w_ treatments + *F. graminearum* inoculation. This was reflected in the total accumulated CO_2_ in the treatments. The maximum DMLs were in the 0.95 a_w_/20–25 °C treatments and at 10 °C/0.95 a_w_. The DMLs were modelled to produce contour maps of the environmental conditions resulting in maximum/minimum losses. Contamination with ZEN/ZEN-related compounds were quantified. Maximum production was at 25 °C/0.95–0.93 a_w_ and 20 °C/0.95 a_w_. ZEN contamination levels plotted against DMLs for all the treatments showed that at ca. <1.0% DML, there was a low risk of ZEN contamination exceeding EU legislative limits, while at >1.0% DML, the risk was high. This type of data is important in building a database for the development of a post-harvest decision support system for relative risks of different mycotoxins.

## 1. Introduction

Zearalenone (ZEN) is a mycotoxin produced by species of *Fusarium graminearum* and *F. culmorum*. While ZEN can be naturally produced in the field in ripening cereals, contamination often occurs post-harvest during storage of feed wheat [1,2]. ZEN has a significant estrogenic effect, especially associated with pig reproduction and can cause loss of embryos and have associated effects on litter size and viability of newborn pigs [3]. There is thus EU legislation on the maximum ZEN contamination levels in food for human consumption (100 μg/kg) and for animal feed (500 μg/kg) [4,5].

The critical control point for minimizing contamination with mycotoxins after harvest is the management of the drying and post-harvest phases. Poor drying immediately after harvesting or storage of damp grain can facilitate the activity of spoilage and mycotoxigenic fungi during the post-harvest phase [6,7,8,9]. This can lead to quality and nutritional losses as well as mycotoxin contamination. For temperate cereals, especially wheat/barley, damp grain can rapidly be colonised by *F. graminearum* resulting in contamination with ZEN and type B trichothecenes. For animal feed, ZEN is of particular concern. There is some data on the ecology of growth and mycotoxin production by strains of *F. graminearum* and *F. culmorum*, predominantly in relation to deoxynivalenol (DON) production [10,11], and in some cases in relation to ZEN production [8]. However, there is no detailed knowledge of the full range of water activity (a_w_) × temperature range for *F. graminearum* colonisation of wheat grain and production of ZEN.

Mylona et al. [8] examined the relationship between respiration of wheat grain colonised by *F. graminearum* and ZEN production, but only at 15–30 °C over a limited a_w_ range. Mylona and Magan [12] showed that it was possible to use respiration rates of *F. langsethiae* colonisation of oats (CO_2_ production) to calculate the relative dry matter losses (DMLs) and relate this to T-2/HT-2 mycotoxin production; this will then be used to determine the relative risks of contamination based on CO_2_ production during storage.

Thus, it is possible to utilise the progressive increase in the respiration rate (R) under increasingly conducive conditions for mould growth due to the oxidation of carbohydrates to produce CO_2_, water vapour and heat during aerobic respiration to calculate quality losses as DML. Overall, a 1% DML corresponds to 14.7 g of CO_2_ produced per kilogram cereal with associated effects on cereal quality [13]. DML can be quantified on the basis of CO_2_ production and respiration rates using Gas Chromatography (GC) [8,9,12] and these data are used as a “storability risk index” to predict overall quality changes in stored grain.

Previously, DML was used as a grain quality indicator with values as low as 0.04% DML being considered to have an impact on seed germination and on early moulding of wheat [14,15]. Seitz et al. [16] showed that a loss of 0.5% DML in stored maize was enough to downgrade this commodity from food to feed grade, with associated increased risks of aflatoxin contamination. Indeed, Mylona et al. [8] showed that DMLs of <1% resulted in maize contaminated with *F. verticillioides* to exceed the EU legislative limits for fumonisins.

The objectives of this study were to examine the effect of storage temperature (T) x water activity (a_w_) conditions (10–25 °C; 0.90–0.95 a_w_) of naturally contaminated, and gamma irradiated stored wheat, or that inoculated with *F. graminearum* on: (a) respiration rate (R), (b) total cumulative CO_2_ production, (c) % DML in the stored wheat treatments, (d) quantification of ZEN contamination levels in the different treatments, and (e) determination of the relationship between DML and ZEN contamination to identify storage conditions which represent a low and high risk of this mycotoxin contamination of wheat during storage.

## 2. Results

### 2.1. Influence of a_w_ and Temperature on the Temporal Respiration Rate and Total CO_2_ Production in Stored Natural and Irradiated Wheat Grain with and without F. graminearum Inoculation

**Figure 1:**
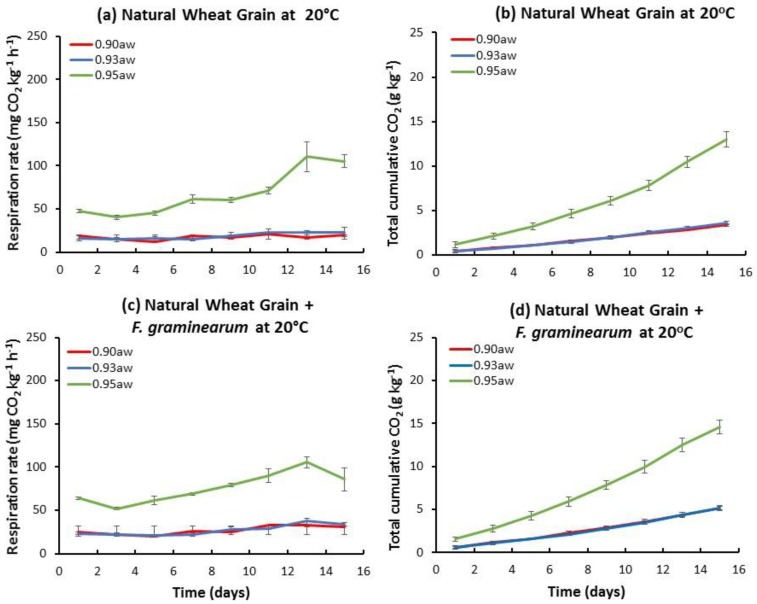
Temporal respiration (**a**,**c**) and total accumulation (**b**,**d**) of CO_2_ by naturally contaminated wheat, and that inoculated with *F. graminearum* stored at 20 °C for up to 15 days at three different water activity levels (a_w_). Vertical bars represent standard errors of the mean (*n* = 4).

**Figure 2:**
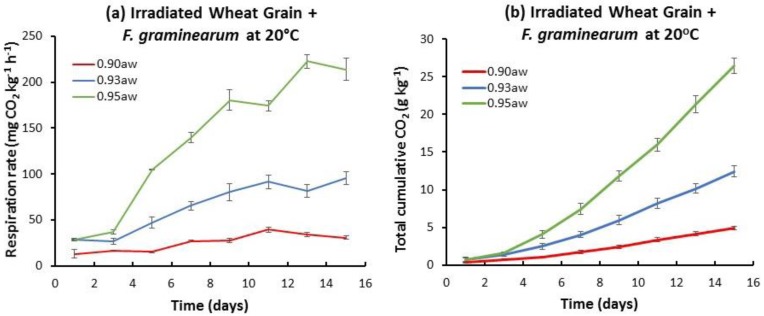
Temporal respiration (**a**) and total accumulation (**b**) of CO_2_ in irradiated wheat inoculated with *Fusarium graminearum* stored at 20 °C for up to 15 days at three different water activity levels (a_w_). Vertical bars represent standard errors of the mean (*n* = 4).

Figure 1 shows an example of the relative temporal CO_2_ production and the total accumulated CO_2_ by the natural mycobiota, or when inoculated with *F. graminearum* at 20 °C in the three a_w_ treatments. The highest respiration rate was at 0.95 a_w_. In the presence of *F. graminearum*, the final accumulated CO_2_ production was slightly higher than that of the naturally contaminated stored wheat grain. A similar pattern was followed in all three a_w_ levels examined. Figure 2 shows the results for gamma irradiated wheat grain inoculated with *F. graminearum*, both in terms of temporal and accumulated CO_2_ production. Both of these parameters show an increase over time with the lowest respiration rates in the 0.90 a_w_ treatment.

Based on these studies at all temperatures and a_w_ levels, the temporal rate and total accumulated CO_2_ production was lowest at 0.90 a_w_ and 10 °C (data not shown). However, at 15 °C there was an increase in respiration, especially at 0.95 a_w_. The cumulative CO_2_ production was overall higher in the irradiated wheat grain + *F. graminearum* treatment. The CO_2_ production data for all treatments were then used to quantify the relative DMLs.

### 2.2. Relationship between Storage Environmental Conditions and Dry Matter Losses (DMLs) in Stored Natural/Irradiated Wheat and That Inoculated with F. graminearum

**Figure 3:**
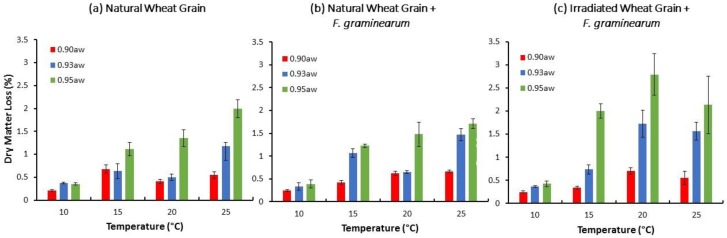
Percentage of Dry Matter Losses (DML) calculated from the total accumulated CO_2_ after 15 days in natural grain (**a**) and natural and irradiated wheat with *F. graminearum* inoculum (**b**,**c**) and incubated at different water activities. Vertical bars represent standard deviation of the mean (*n* = 4).

Figure 3 compares the DMLs in natural/irradiated stored wheat grain with and without *F. graminearum* inoculation after 15 days storage. DML increased with increasing a_w_ and T (*p* < 0.05) (Supplementary Table S1). Inoculation with *F. graminearum* always resulted in an increase in the DML in all storage treatment conditions, with the exception of natural wheat at 25 °C/0.95 a_w_. Maximum DMLs were approx. 2% and 3% in natural/irradiated wheat inoculated with *F. graminearum*, respectively.

A polynomial model

(Log_10_DML = b_0_ + b_1_T + b_2_a_w_ + b_3_a_w_ × T), (1)

was obtained by forward stepwise regression for the effect of the storage conditions on the losses in the log_10_ transformed data of DMLs in natural wheat and in natural wheat colonized by *F. graminearum* (r^2^ = 0.81). However, a_w_ × T was not included in the model since the interaction was not statistically significant. The actual values for the three coefficients and the r^2^ adjusted in the natural stored grain were: b_0_ = −8.22 ± 1.00, b_1_ = 0.03 ± 0.00, b_2_ = 8.04 ± 1.09, r^2^ = 0.71; and b_0_ = −8.01 ± 0.76, b_1_ = 0.03 ± 0.00, b_2_ = 7.99 ± 0.81, r^2^ = 0.82 for natural wheat and that inoculated with *F. graminearum*, respectively. This was b_0_ = −12.47 ±1.00, b_1_ = 0.04 ± 0.00, b_2_ = 12.60 ± 1.09, r^2^ = 0.87 for irradiated wheat inoculated with *F. graminearum.*

**Figure 4:**
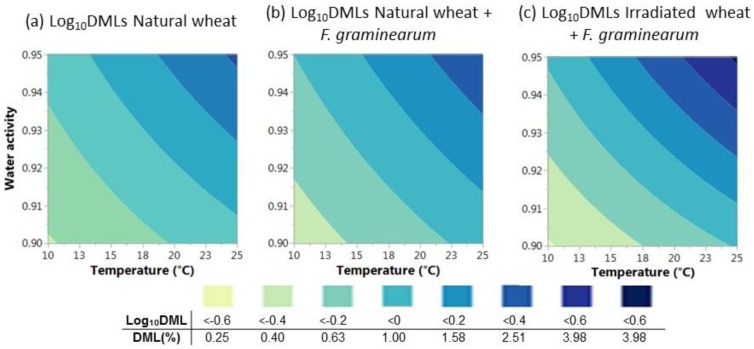
Percentage of Dry Matter Losses contour maps describing the DMLs in natural grain (**a**,**b**) and irradiated wheat (**c**) inoculated with *F. graminearum* under different combinations of environmental conditions.

Figure 4 shows that the DMLs increased in the same environmental combination in natural wheat due to *F. graminearum* colonization. DMLs > 1% occurred with T > 14 °C in natural wheat, however; the same DMLs in the presence of *F. graminearum* occurred at lower Ts (10 °C/0.95 a_w_).

### 2.3. Zearalenone and Its Associated Compounds in Stored Wheat Inoculated with F. graminearum

Table 1 shows the ZEN and associated compounds isolated from the naturally stored wheat grain and the natural and irradiated wheat grain treatments inoculated with *F. graminearum* and stored for 15 days. Temperature significantly (*p* < 0.05) affected the ZEN contamination, while a_w_ was only significant in stored naturally contaminated wheat (see Supplementary Table S1). A similar trend was observed for the derivatives, alpha-zearalenone, beta-zearalenone and zearalenone-sulfate found in these stored wheat treatments.

### 2.4. Correlation between DMLs and ZEN Contamination Relevant to EU Legislative Limits

**Figure 5:**
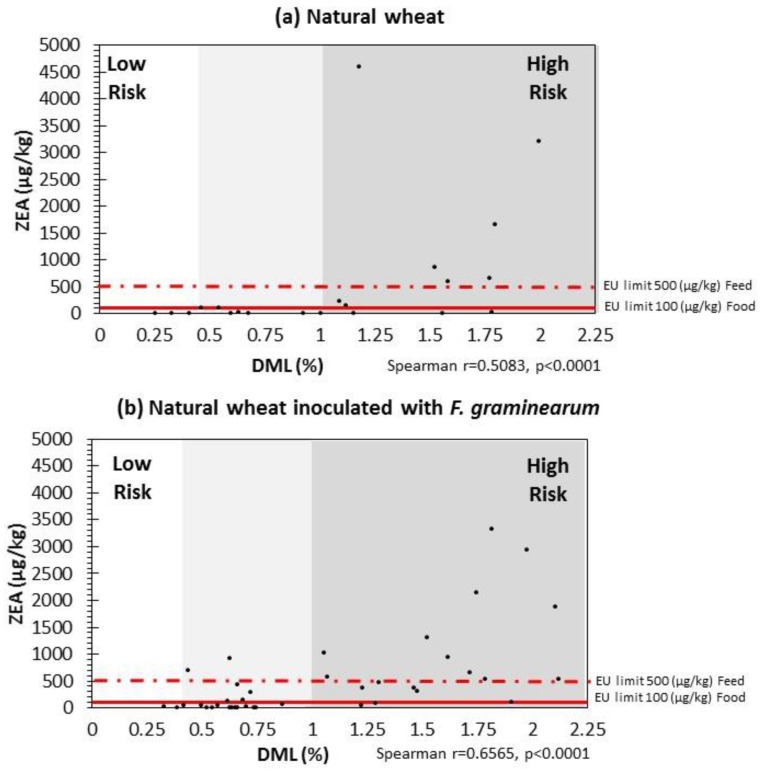
Scatter-plot of Dry Matter Losses (DMLs) and Zearalenone in stored wheat after 15 days storage under all the environmental conditions examined producing by natural mycobiota (**a**) and both natural mycobiota and *F. gramineraum* (**b**). The red lines indicate the EU legislative limits for unprocessed wheat for human consumption (solid line) and animal feed (dash line) (EC, 576/2006, 1881/2006).

ZEN data was subsequently plotted against DMLs for natural wheat, and natural/irradiated wheat inoculated with *F. graminearum* in Figure 5. This also has indicator lines depicting the EU legislative limits for ZEN in unprocessed cereals (100 µg/kg) and for animal feed (500 µg/kg) [4,5]. This shows that very small changes in DML can result in the legislative limits being exceeded. In natural grain, 1% DML represents the relative change from low to higher risk. For stored natural wheat grain with *F. graminearum*, the DML loss level, before exceeding the limit, was actually lower 0.5–0.75% DML. In irradiated wheat this was closer to 1.0–1.25% DML. Overall, a DML of ca. 1% could thus be set as an index of risk with a higher probability of exceeding the legislative limits for ZEN in stored wheat. These results suggest that to minimise the risk of ZEN, storage at ≤12 °C and <0.93 a_w_ is necessary for wheat.

## 3. Discussion

This study compared the effect of storage environment and colonization by naturally present mycobiota of wheat or that inoculated with a strain of *F. graminearum* to examine the relationship between respiration activity/DMLs and ZEN contamination. There was a consistent link between rates of CO_2_ production and the storage moisture and temperature conditions. This is important as any delays in drying of relatively moist cereals (20–25% MC = 0.90–0.95 a_w_) would allow *Fusarium* species to colonise the grain rapidly causing both quality loss and contamination with ZEN and type B trichothecenes [8,17,18,19,20]. There has also been a lack of knowledge on the growth boundary conditions for *F. graminearum* with most studies focused on parameters >0.95 a_W_ [2,21,22]. Previously, it was shown that in irradiated maize grain, *F. graminearum* was able to grow at between 10–35 °C at 0.97 a_w_ [23], but not at 37 °C even with freely available water (0.99 a_w_) after 11 days storage [24].

With irradiated “clean” wheat grain with retained germinative capacity, *F. graminearum* colonization was relatively rapid, resulting in a significantly higher DML observed, especially at 20–25 °C and 0.95 a_w_. The different DMLs observed in the naturally contaminated wheat grain and that with additional *F. graminearum* inoculum showed that the former treatment had a higher loss in quality. This may be due to the colonization of the wheat by a range of different species which make up the fungal community which can produce a battery of enzymes to break down the wheat nutrients. The presence of additional *F. graminearum* inoculum could influence interactions with resident mycobiota and this competition may have an impact on the ability to degrade the wheat nutrients as efficiently. Under the range of conditions examined, predominantly mesophilic fungi will be involved in colonizing the stored wheat grain [6]. Their interactions have previously been shown to also influence mycotoxin production in stored wheat [7].

As found previously in oats (*F. langsethiae*) and maize (*F. verticillioides*), DML was significantly influenced by a_w_ and temperature. However, in recent studies with paddy and brown rice and *F. verticillioides* and fumonisin contamination, temperature alone was not a significant factor, although a_w_ and a_w_ × temperature interactions were. Only 25 and 30 °C were used in these studies which are in the range for optimum growth of this mycotoxigenic species [25]. Furthermore, the difference in DML between paddy and brown rice was significant, regardless of whether the rice types were stored with natural mycobiota or with additional inoculum of *F. verticillioides*. In paddy rice, the maximum DML was only about 2.0–3.0% (0.95 a_w_/25–30 °C) while in brown rice this was 12–16% under the same environmental conditions after 10 days storage. In the present study, the DMLs varied from 0.25% at 10 °C to 3.0–3.5% at 20–25 °C, especially at 0.95 a_w_.

In the stored wheat, the ZEN production was highest at 25 °C and 0.95 and 0.93 a_w_ and at 20 °C and 0.95 a_w_. The related secondary metabolites, alpha-ZOL and beta-ZOL, appeared to be produced optimally under these similar storage conditions. However, there was usually more of the latter compound present. For ZEN, very little was produced at 10 °C regardless of a_w_. This certainly suggests that 20–25 °C represents a particular risk for production of ZEN, provided a_w_ conditions are conducive (>0.93 a_w_). Previous studies by Mylona et al. [8] showed that at 30 °C there was a significant reduction in the production of ZEN in stored wheat grain indicating that intermediate temperatures of 15–25 °C may be more important in relation to ZEN contamination of cereal-based commodities.

Some previous studies have focused on ZEN production in maize infected with *F. graminearum*. For example, ZEN production by this species on maize was reported at 10 to 35 °C [23]. Several studies have suggested that fungal growth and ZEN production increased with the moisture content (m.c.) of the substrate [26,27,28]. However, other studies suggest that very low temperatures and relative humidities inhibit ZEN production [29]. Recently, Lahouar et al. [20] reported optimum ZEN production values at 0.91 and 0.94 a_w_ after two weeks storage in sorghum grain. Indeed, in the present study a_w_ was not a significant factor when relatively high amounts of ZEN was present in wheat at 0.90 a_w_ in the irradiated wheat grain inoculated with *F. graminearum* after 15 days storage.

The relationship between DML and ZEN production was examined in the context of the EU legislative limits for this mycotoxin in food and feed. This certainly suggests that where you have naturally contaminated wheat under the conditions examined, a DML of >1% would represent a high risk of ZEN levels above the legislative limits. Where there is a higher inoculum of *F. graminearum*, this could be even less and between 0.5–0.75% DML. Above this range the risk is significantly increased. Previous studies with DML and DON, and with *F. verticillioides* and fumonisins in maize suggested that at between 0.8–1% DML, there is an increased risk of contamination levels exceeding the limits set by the EU [8].

In conclusion, CO_2_ production appears to be a good indicator of relative quality of grain when coming into store and whether it might be at a m.c. which is conducive to colonisation by mycotoxigenic fungi and mycotoxin contamination. This also allows comparison with respiration rates at 0.70 a_w_ (=14.5–15% m.c.) which represents safe storage conditions. For wheat, the production is about 5–6 mg CO_2_/kg/hr. In addition, potential exists for using the respiration rates and related DMLs to provide an indication of the relative likelihood of mycotoxin contamination. This type of information could be effectively used as part of a Decision Support System (DSS) specifically for better post-harvest management of such cereals to minimise mycotoxin contamination and allow rapid remedial action to be taken.

## 4. Materials and Methods

### 4.1. Fungal Culture

*F. graminearum* strain Fg 08/111 isolated from UK wheat, and a known producer of ZEN and DON was used in this study. This was kindly supplied by Prof. S. Edwards, Harper Adams University, Shropshire, U.K. Cultures of this strain were maintained in glycerol:water (67:33) at −20 °C and sub-cultured when required for experimental use.

### 4.2. Wheat Grain Treatment, Moisture Content and Water Activity Adjustments

Natural wheat grain or wheat grain which was gamma irradiated with 12–15 kGry was used in these studies. The latter grain had retained germinate capacity but was free of contaminating microorganism. Both grain treatments were used to build adsorption moisture retention curves for carrying out storage experiments. 10 g sub-samples were placed in 25 mL Universal bottles and known amounts of water added, and the sub-samples sealed and stored at 4 °C for 24 h with regular shaking. The samples were then equilibrated at 25 °C and the water activity (a_w_) and m.c. determined. The a_w_ was measured using a AquaLAB Water Activity Meter 4 TE (Decagon Devices, Inc., Pullman, WA, USA) at 25 °C. The a_w_ of the natural unmodified wheat grain was 0.70 and for the irradiated grain 0.76 a_w_. The moisture content (wet weight basis) was also determined by drying at 105 °C for 17 h. The amounts of added water were plotted against a_w_ levels to help accurately modify the grains to the target a_w_ treatment levels. 

### 4.3. Grain Inoculation and Incubation

Wheat grain sub-samples (10 g) were modified to different a_w_ levels with water/sterile water (0.90, 0.93 and 0.95 a_w_) and equilibrated as detailed previously in 40 mL vials (Chromacol Ltd., Welwyn, UK) with sealable caps containing a septum for gas removal. The initial a_w_ was checked at the beginning and end of each experiment. The treatments and replicates were stored in 10 L environmental chambers which contained glycerol-water solutions (2 × 450 mL) in beakers to maintain the equilibrium relative humidity (ERH) of the atmosphere at the target a_w_ levels of the stored wheat samples (10–25 °C). Glycerol solutions were renewed weekly. Four 5 mm diameter agar disks were taken from a 7 day old colony of the *F. graminearum* strain grown on V8 agar (V8^®^, 175 mL; CaCO_3_, 3 g; ZnSO_4_·7H_2_O, 0.01 g; CuSO_4_·5H_2_O, 0.005 g; agar, 20 g/L) and transferred to vials containing the different natural and irradiated wheat treatments and replicates and mixed thoroughly. Control treatments without inoculum were also carried out in parallel. The vials were kept open in the storage chambers and only sealed prior to CO_2_ analyses. In all cases, four replicates per treatment were used.

### 4.4. Respiration Measurement by Gas Chromatography (GC)

Carbon dioxide (CO_2_) production was measured on alternate days (1, 3, 5, 7, 9, 11, 13 and 15). The sampling method used was based on that detailed previously [30] but the specific head space volume was considered. For calculating the head space, vials containing the modified grain was quantified. The head space volumes were 34, 33 and 32 mL for 0.90, 0.93 and 0.95 a_w_ respectively.

Vials were sealed under sterile conditions and stored for 1 h at the treatment conditions before CO_2_ was quantified. Five mL of the headspace was withdrawn and 2 mL was directly inserted into the sampling chamber of the GC for CO_2_ analysis. The GC equipment used was an Agilent 6890N Network Gas Chromatograph (Agilent Technologies, Cheshire, UK) with a Thermal Conductivity Detector (TCD) and helium as a carrier gas. The column used for the analysis was packed with Chromosorb 103 and the data were analysed using Agilent Chemstation Software (Agilent Technologies, Cheshire, UK). Calibration standard used was 10.06% CO_2_, 2% O_2_ in nitrogen (BOC cylinder).

The percentages of CO_2_ concentration were used to calculate the Respiration (R) in mg CO_2_ /(kg h), total cumulative production of CO_2_ and the total Dry Matter Loss (DMLs) [30].

### 4.5. Mycotoxin Analyses

#### 4.5.1. Sample Preparation

Grain was dried for 48 h at 60 °C, milled and stored at 4 °C until analyses. Five grams of each milled sample were extracted using 20 mL extraction solvent (acetonitrile-water-acetic acid, 79:20:1, *v*/*v*/*v*) followed by a 1+1 dilution using acetonitrile-water-acetic acid (20:79:1, *v*/*v*/*v*) and direct injection of 5 µL diluted extract into the sampling port for LC-MS/MS analysis.

#### 4.5.2. LC-MS/MS Parameters

Liquid chromatography-tandem mass spectrometry (LC-MS/MS) screening of target fungal metabolites was performed with a QTrap 5500 LC-MS/MS System (Applied Biosystems, Foster City, CA, USA) equipped with a TurboIon Spray electrospray ionization (ESI) source and a 1290 Series HPLC System (Agilent, Waldbronn, Germany). Chromatographic separation was performed at 25 °C on a Gemini^®^ C_18_-column, 150 × 4.6 mm i.d., 5 µm particle size, equipped with a C_18_ 4 × 3 mm i.d. security guard cartridge (all from Phenomenex, Torrance, CA, USA). The chromatographic method as well as chromatographic and mass spectrometric parameters are described in Malachova et al. [31,32] and allows the detection of 295 metabolites. Electrospray ionization-tandem mass spectrometry (ESI-MS/MS) was performed in the time-scheduled multiple reaction monitoring (MRM) mode both in positive and negative polarities in two separate chromatographic runs per sample by scanning two fragmentation reactions per analyte. The MRM detection window of each analyte was set to its expected retention time ±27 s and ±48 s in the positive and the negative mode, respectively. Confirmation of positive analyte identification was obtained by the acquisition of two MRMs per analyte (with the exception of moniliformin, MON, and 3-nitropropionic acid that exhibit only one fragment ion), which yielded 4.0 identification points according to commission decision 2002/657/EC. In addition, the LC retention time and the intensity ratio of the two MRM transitions agreed with the related values of an authentic standard within 0.1 min and 30% rel., respectively.

Quantification was performed via external calibration using serial dilutions of a multi-analyte stock solution. For ZEN and its metabolites, the validated recoveries were 103.9%, for zearlalenone-suphate (100%), zearalenol (91.1%). The limit of detection were 0.12 µg/kg and 0.8 µg/kg for ZEN and ZOL, respectively. The accuracy of the method has been verified on a continuous basis by regular participation in proficiency testing schemes [31,32]. This approach was recently used to examine the effect of interacting environmental factors on targeted metabolomics profiles in stored naturally contaminated wheat grain and that inoculated with *F. graminearum* [33].

### 4.6. Statistical Analysis and Modelling the Results

Statistical analysis was performed using the package JMP^®^ Pro 13 (SAS Institute Inc., 2016, Cary, NC, USA). Datasets were tested for normality and homoscedasticity using the Shapiro-Wilk and Levene test, respectively. When data failed the normality test, variable transformation was performed to try to improve normality or homogenise the variances. Transformed data were still not normally distributed and therefore the Wilconxon or Kruskal-Wallis test by ranks was used for the analysis of the data. Nonparametric comparisons for each pair using the Wilcoxon Method were used to find differences between groups.

Forward stepwise regression was used to obtain polynomial equations for Log_10_DML with regard to the storage conditions (a_w_ and T). The assumptions of linearity and normally distributed residual were assessed, producing normal plots of the residuals. Contour maps were built in JMP^®^ Pro 13 using 5000 simulation data from the predicted formula.

The Spearman rank order correlations test was used to determine the significance of the correlation between the variables since some variables were not normally distributed.

## Figures and Tables

**Table 1:** Zearalenone and their metabolites produced by *F. graminearum* in different wheat treatments under different environmental conditions after 15 days storage.

		Natural Wheat			Natural Wheat + *F. graminearum*			Irradiated Grain + *F. graminearum*		
T (°C)	a_w_	ZEN^1^	alpha-ZOL^2^	beta-ZOL^3^	ZEN^1^	alpha-ZOL^2^	beta-ZOL^3^	ZEN^1^	alpha-ZOL^2^	beta-ZOL^3^
10	0.90	<LOD	<LOD	<LOD	<LOD	<LOD	<LOD	<LOD	<LOD	<LOD
10	0.93	6.0	<LOD	<LOD	25.5	<LOD	<LOD	1.1	<LOD	<LOD
10	0.95	<LOD	<LOD	<LOD	1.0	<LOD	<LOD	0.9	<LOD	<LOD
15	0.90	16.7	<LOD	<LOD	195.9	2.6	12.0	16.7	<LOD	3.3
15	0.93	18.5	1.1	3.6	551.8	2.7	7.4	15.1	<LOD	3.3
15	0.95	131.9	1.2	6.7	241.9	2.9	8.2	22.1	<LOD	6.3
20	0.90	0.5	<LOD	<LOD	110.9	<LOD	3.2	120.8	2.2	24.6
20	0.93	1.0	<LOD	<LOD	11.4	<LOD	<LOD	178.4	2.5	43.0
20	0.95	777.5	13.3	51.9	810.2	9.1	35.7	219.8	4.9	78.7
25	0.90	112.5	2.8	16.3	382.6	8.5	16.4	1608.7	13.2	246.6
25	0.93	1536.9	19.9	84.9	1489.7	13.3	81.5	633.7	6.2	83.7
25	0.95	1167.8	15.6	59.7	1461.4	11.4	78.3	1030.2	10.2	227.0

^1^ Zearalenone, ^2^ alpha-Zearalenol, ^3^ beta-Zearalenol (µg/kg) <LODZEA = 0.12 µg/kg and <LODalpha-Zol and <LODbeta-Zol = 0.8 µg/kg. Recoveries were 103.9%, for zearlalenone-suphate (100%), zearalenol (91.1%). Results have been corrected respectively for recoveries. Maximum SE Natural wheat: 1080.45, 4.67 and 27.83 µg/kg; 566.85, 4.99 and 23.25 µg/kg and 162.28, 1.56 and 18.19 µg/kg for natural wheat, natural wheat + *F. gramineraum* and irradiated grain + *F. graminearum*. Only positive values were included in the analysis. Within columns the heat maps show that the red and amber are higher concentrations than the yellow treatments.

## Supplementary Materials

The following are available online at http://www.mdpi.com/2072-6651/10/2/86/s1. Table S1. Statistical results.
