# Supplementary material for: Fusarium graminearum in Stored Wheat: Use of CO2 Production to Quantify Dry Matter Losses and Relate This to Relative Risks of Zearalenone Contamination under Interacting Environmental Conditions
Source: Toxins (Basel). 2018 Feb 17;10(2):86. doi: 10.3390/toxins10020086 (PMC5848187; doi:10.3390/toxins10020086)
Supplement: Supplementary file 1 [file toxins-10-00086-s001.pdf]

Table S1. Supplementary Materials: Fusarium graminearum in Stored Wheat: Use of CO2 Production to Quantify Dry Matter Losses and Relate This to Relative Risks of Zearalenone Contamination under Interacting Environmental Conditions

Esther Garcia-Cela, Elsa Kiaitsi, Michael Sulyok, Angel Medina and Naresh Magan

Table S1. Statistical results p-values.

		Wilconxon/Kruskal-Wallis Tests (Rank Sums)				Nonparmetric Comparisons for each pair using Wilcoxon Method				
		**DML**	**ZEN**	**alpha-ZOL**	**beta-ZOL**	**Level - Level**	**DML**	**ZEN**	**alpha-ZOL**	**beta-ZOL**
Natural wheat	**T**	<.0001	0.0081	0.0175	0.0333	**25-10**	<.0001	0.0013	0.0071	0.0071
						**15-10**	0.0002	0.0195	0.0424	0.0786
						**20-10**	0.0005	0.0722	0.1217	0.0788
						**25-15**	0.126	0.0685	0.1662	0.0788
						**25-20**	0.2855	0.1821	0.1662	0.2454
						**20-15**	0.665	0.777	0.8939	0.9395
	**a_w**	0.001	0.0326	0.1043	0.0188	**0.95-0.9**	0.0011	0.0134	0.0785	0.0261
						**0.95-0.93**	0.0031	0.0909	0.1073	0.0261
						**0.93-0.9**	0.2828	0.3618	1	1
Natural wheat + *F. graminearum*	**T**	<.0001	<.0001	0.0007	<.0001	**25-10**	<.0001	<.0001	0.0004	<.0001
						**15-10**	<.0001	<.0001	0.0123	0.0002
						**20-10**	0.0011	0.0001	0.0191	0.0022
						**25-15**	0.0226	0.012	0.0286	0.0051
						**25-20**	0.665	0.0247	0.0788	0.0165
						**20-15**	0.795	0.3401	0.4967	0.1725
	**a_w**	0.0003	0.2301	0.0516	0.1126	**0.95-0.9**	0.0004	0.0791	0.0153	0.0335
						**0.95-0.93**	0.0056	0.4151	0.1377	0.2628
						**0.93-0.9**	0.0365	0.5012	0.3753	0.5306
Irradiated grain wheat + F. *graminearum*	**T**	0.0001	<.0001	<.0001	<.0001	**25-10**	<.0001	<.0001	<.0001	<.0001
						**15-10**	0.0003	<.0001	<.0001	<.0001
						**20-10**	0.0141	<.0001	<.0001	<.0001
						**25-15**	0.1166	<.0001	0.0001	<.0001
						**25-20**	0.1572	<.0001	0.0001	0.0004
						**20-15**	0.8777	<.0001	1	0.0029
	**a_w**	<.0001	0.9325	0.9603	0.6707	**0.95-0.9**	0.0002	0.6647	0.6978	0.2729
						**0.95-0.93**	0.0005	0.8652	1	0.8025
						**0.93-0.9**	0.0135	0.8651	0.9837	0.8922

Red numbers $p<0.01$. Orange numbers $p<0.05$. Only positives samples were used in the analysis.
